# Indocyanine green fluorescence in robot-assisted minimally invasive esophagectomy with intrathoracic anastomosis: a prospective study

**DOI:** 10.1007/s13304-022-01329-y

**Published:** 2022-08-17

**Authors:** E. M. de Groot, G. M. Kuiper, A. van der Veen, L. Fourie, L. Goense, S. van der Horst, J. W. van den Berg, R. van Hillegersberg, J. P. Ruurda

**Affiliations:** Department of Surgery, University Medical Center Utrecht, POBOX 85500, 3508 GA Utrecht, The Netherlands

**Keywords:** RAMIE, Fluorescence, Indocyanine green, Esophagectomy, Anastomotic leakage

## Abstract

Indocyanine green fluorescence angiography (ICG-FA) allows for real-time intraoperative assessment of the perfusion of the gastric conduit during esophagectomy. The aim of this study was to investigate the effect of the implementation of ICG-FA during robot-assisted minimally invasive esophagectomy (RAMIE) with an intrathoracic anastomosis. In this prospective cohort study, a standardized protocol for ICG-FA was implemented in a high-volume center in December 2018. All consecutive patients who underwent RAMIE with an intrathoracic anastomosis were included. The primary outcome was whether the initial chosen site for the anastomosis on the gastric conduit was changed based on ICG-FA findings. In addition, ICG-FA was quantified based on the procedural videos. Out of the 63 included patients, the planned location of the anastomosis was changed in 9 (14%) patients, based on ICG-FA. The median time to maximum intensity at the base of the gastric conduit was shorter (25 s; range 13–49) compared to tip (34 s; range 12–83). In patients with anastomotic leakage, the median time to reach the FImax at the tip was 56 s (range 30–83) compared to 34 s (range 12–66) in patients without anastomotic leakage (*p* = 0.320). The use of ICG-FA resulted in an adaptation of the anastomotic site in nine (14%) patients during RAMIE with intrathoracic anastomosis. The quantification of ICG-FA showed that the gastric conduit reaches it maximum intensity in a base-to-tip direction. Perfusion of the entire gastric conduit was worse for patients with anastomotic leakage, although not statistically different.

## Introduction

Esophagectomy, generally combined with neoadjuvant therapy, is the cornerstone of curative treatment for esophageal cancer [1]. Anastomotic leakage remains one of the most feared postoperative complications following esophagectomy and occurs in approximately 10–30% of the patients [2]. It has been associated with severe morbidity, mortality and increase in healthcare costs [3, 4]. Finding new techniques to reduce anastomotic leakage rates is, therefore, of high priority.

The etiology of anastomotic leakage is multifactorial and includes patient factors such as diabetes mellitus, smoking status and generalized vascular disease [5, 6]. The higher prevalence of anastomotic leakage in these patients is hypothesized to result from hypoperfusion of the gastric conduit [7, 8]. Consequently, intraoperative evaluation of the anastomosis to ensure adequate vascular perfusion may reduce the risk of anastomotic leakage.

Indocyanine green fluorescence angiography (ICG-FA) has been increasingly considered as a tool for real-time intraoperative assessment of tissue perfusion. After intravenous injection, indocyanine green is distributed throughout the systemic blood circulation, emitting a green fluorescent signal visible in vascularized tissue. The timing and intensity of the fluorescent signal can potentially provide useful information on both vascular supply as well as microperfusion of the gastric conduit [9].

The aim of this prospective study was to evaluate the effect of the implementation of ICG during robot-assisted minimally invasive esophagectomy (RAMIE) with intrathoracic anastomosis.

## Methods

### Patients

A standardized protocol for ICG fluorescence as a means of determining the gastric conduit perfusion was implemented in the UMC Utrecht in December 2018. All consecutive patients who underwent RAMIE with an intrathoracic anastomosis were included. Treatment details were prospectively recorded in the institutional database. The institutional review board of the hospital approved this study and the need for informed consent was waived.

### RAMIE procedure

**Fig. 1:**
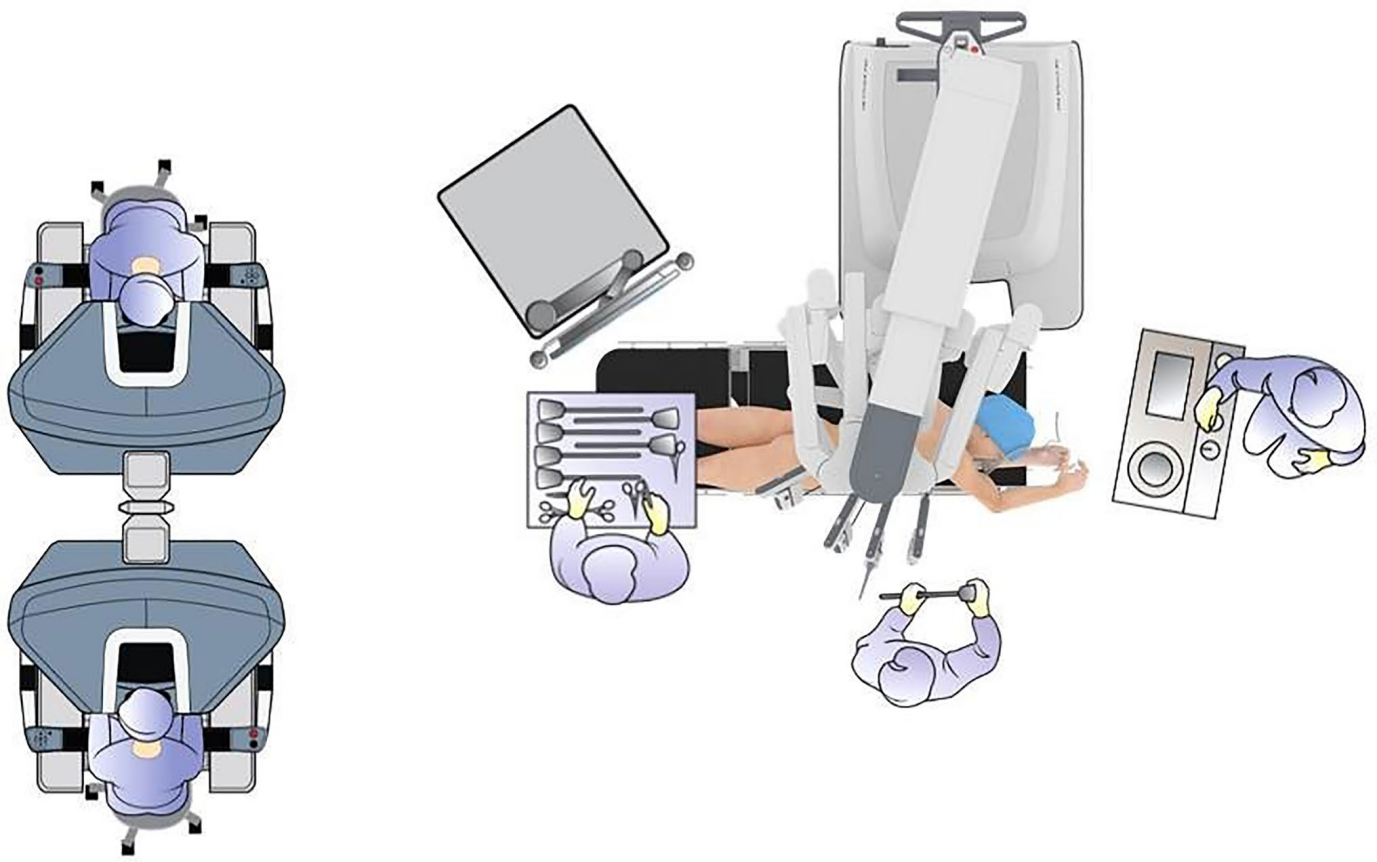
Positioning of the patient and robotic system during robot-assisted minimally invasive esophagectomy

All patients underwent full RAMIE with robotic abdominal and thoracic phase. For the thoracic phase, patients were placed in a semiprone position (Fig. 1). A hand-sewn robot-assisted intrathoracic anastomosis was performed in all patients. The procedure and the anastomotic technique have been described in detail previously [10, 11]. In summary, an end-to-side construction was performed to create the anastomosis. The posterior and anterior walls were closed with a single-layer, running, barbed V-Loc 4.0 suture. Hereafter, 3 to 4 tension releasing stitches were placed to balance the tension on the anastomosis. Finally, an omental wrap was positioned around the anastomosis. All surgeries were performed by JR and RvH who both completed the learning curve for RAMIE [12].

### Indocyanine green angiography and video recording

**Fig. 2:**
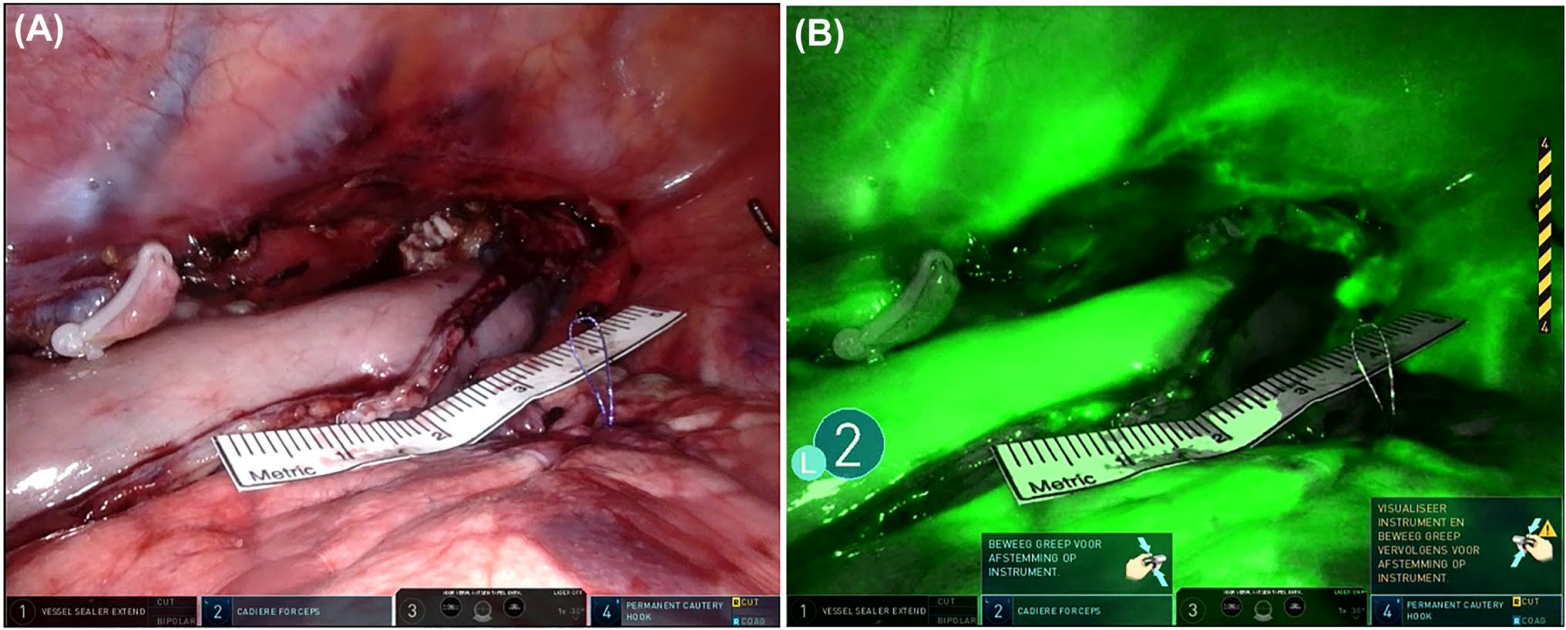
Camera view of surgical site with the robotic camera on normal 3DHD illumination mode **a** and on FireFly Fluorescence Imaging mode **b**

ICG was applied before the creation of the anastomosis during the thoracic phase of RAMIE. Prior to administration of ICG, the surgeon determined the location of the anastomosis with an intracorporeal ruler and reported the distance measured from the tip of the gastric conduit in centimeters. Hereafter, the robotic camera on the da Vinci XI system (Intuitive Surgical, Sunnyvale, CA) was switched from normal 3DHD Illumination mode to the Firefly Fluorescence Imaging mode. The robotic camera was positioned in the thoracic cavity, aimed at the gastric conduit, providing a clear view of the entire gastric conduit surface as well as the visible part of the right lung and thoracic wall, including the embedded intercostal arteries (Fig. 2). Both lung and thoracic wall are known to be well-vascularized structures and were, therefore, used as reference points, the lung for venous vascularization and the thoracic wall arteries for arterial blood supply. The distance between the camera and the gastric conduit was not standardized as individual patient anatomy had to be taken into account. Subsequently, 7.5 mg ICG was administered via a peripheral intravenous cannula. After injection, the robotic camera was fixed for 90 s. The fixed camera is important as the measurement of the fluorescent signal becomes inaccurate with any movement of the image. This inaccuracy is caused by the inverse correlation between fluorescence intensity and camera distance, therefore, movement of the camera would cause disturbance of the measurement [13]. After 90 s, the surgeon reported whether the initial chosen site for the anastomosis on the gastric conduit was changed based on ICG-FA. If that was the case, the distance measured from the final location of the anastomosis to the tip of the gastric conduit was reported.

### Analysis of ICG videos

Processing and analysis of the videos was performed with the software program ImageJ (National Institutes of Health, Maryland, US). ImageJ enables selection of multiple regions of interest (ROI) on images after which fluorescence intensity can be measured in each of these regions separately. The fluorescence intensity is measured in means of greyscale, hence, enhancement of any color will be detected. With ICG, green is the only color on the footage as the *FireFly* Fluorescence Imaging mode on the da Vinci XI shows all colors except for the fluorescent signal in black and white.

**Fig. 3:**
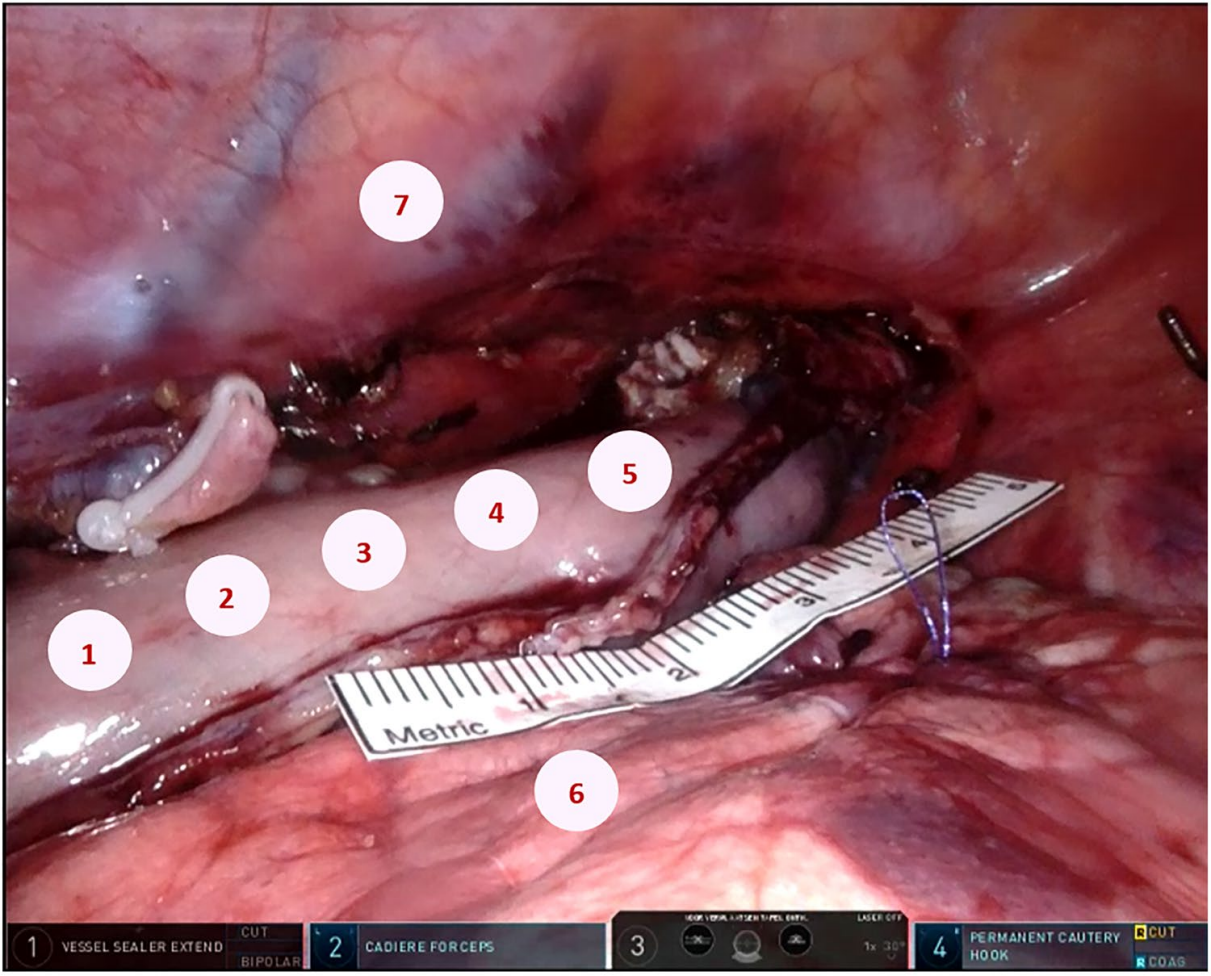
Image of the surgical site with regions of interest placed at the base **1**, on ¼ of the conduit from base towards the tip **2**, on ½ of the conduit from base towards the tip **3**, on ¾ of the conduit from base towards the tip **4**, at the tip of the conduit **5** and at the right lung **6** and the thoracic wall **7**

First, screen captures were made of the ICG with a one-second interval, facilitating a one frame-per-second analysis of the recorded videos resulting in 90 images per patient to be analyzed. Second, 7 fixed ROI were placed on individual images. Five ROI were placed on the gastric conduit: at the base, on ¼ of the conduit from base towards the tip, on ½ of the conduit from base towards the tip, on ¾ of the conduit from base towards the tip and at the tip of the conduit. The base of the conduit was defined as the site at the level of the hiatus and the tip as the end of the conduit at the level of the thoracic aperture, as demonstrated in Fig. 3. The two remaining ROI were placed on the right lung and the thoracic wall, serving as reference points (Fig. 3). Last, the mean gray value of each ROI on every image was measured, resulting in the objectively quantified fluorescence intensity of five parts of the gastric conduit, the lung, and the thoracic wall at every second during the ICG procedure.

**Fig. 4:**
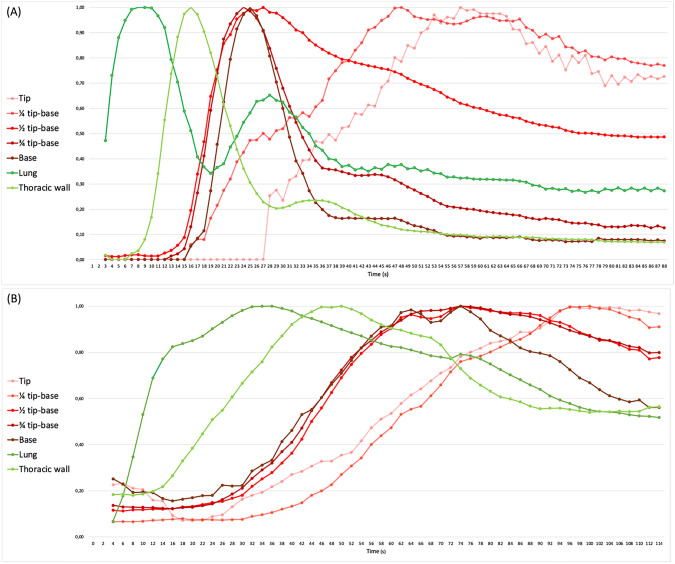
Time–intensity curves of different ROIs on the gastric conduit, the lung and the thorax in a patient undergoing RAMIE, showing a ‘normal’ curve **a** and ‘delayed’ curve **b**. The time to maximum fluorescence (wash-in), i.e., the arterial inflow, is reflected by the increasing part of the graph, until the maximum intensity is reached. The wash-out time of fluorescent dye, i.e., the venous outflow, is reflected by the interval from the up rise of a wave to the time when rapid fall of signal intensity was changed to slow downfall. In **a**, the thoracic wall, lung and the base of the gastric conduit show a quick wash-in and wash-out line, while the tip of the gastric conduit shows an impaired wash-in and wash-out time. This could be explained by the fact that the vascularization of the tip of the gastric conduit is dependent on intramural perfusion while the base of the conduit is directly vascularized by the right gastroepiploic artery

After video analysis, the collected data were plotted in time–fluorescence intensity curves, with time in seconds on the *X*-axis. An example of this curve is shown in Fig. 4. The *Y*-axis represents relative fluorescence intensity of each ROI calculated using the formula $\frac{{{\text{FI at }} t (x) }}{{ {\text{FI}}_{\rm max} }}$, with FI_max_ being the maximum fluorescence intensity reached at the ROI throughout the 90-s time span. This formula corrects for the varying distance between the camera and the individual ROI, as absolute FI-values are not representative due to the inverse correlation with camera distance. To account for the heart rate and respiratory rate causing pulsation and movement of the tissue in the placed ROI, a moving average with an interval of 3 s was used for the *Y*-values. In this graphical depiction of fluorescence intensity of several ROI over time, a time to maximum FI can be established (*T*_max_).

### Outcomes

The primary outcome consisted of the difference between the planned location of the anastomosis before and after ICG-FA. This outcome was prospectively collected with a case report form. The secondary outcome was the time to maximum fluorescence intensity measured in 7 regions of interests (ROIs); 5 ROIs on the gastric conduit from the base to the tip and 1 on the lung and thoracic wall, respectively. Patients were excluded if the surgical videos were missing or if the recording of ICG-footage was not performed according to the protocol. The initial enhancement of the lung was used as starting time. The maximum intensity was measured in gray scale which is described below. The time to maximum fluorescence intensity was demonstrated for patients with and without anastomotic leakage. Anastomotic leakage was defined according to the Esophagectomy Complications Consensus Group classification [14].

### Statistical analysis

Categorical variables were shown as a number with percentage. Continuous data were reported as mean with standard deviation or median with range, pending on data distribution. Categorical variables were compared using the Chi-square test. The Fisher’s exact test was used in case of small cell counts. Continues variables were compared using the Mann–Whitney *U* test. *P* < 0.05 were considered statistically significant. All statistical analyses were performed using SPSS 25.0 (IBM).

## Results

### Patients

Between 2018 and 2022, 67 patients underwent RAMIE with intrathoracic anastomosis of which 4 were excluded. In 2 patients, the case report form was missing and in 2 patients ICG-FA was not performed due to conversion to thoracotomy and saturation problems. Consequently, 63 patients were included in the study. Baseline characteristics are shown in Table 1. The majority was treated with neoadjuvant therapy (*n* = 57, 91%). Out of 63, 26 (41%) patients suffered from vascular comorbidity and 13 (21%) patients had diabetes mellitus. Anastomotic leakage occurred in 14 (22%) patients which consisted of a grade I in 3 patients, grade II in 8 patients and grade III in 3 patients.

**Table 1:** Patients’ characteristics of 63 patients who underwent RAMIE with intrathoracic anastomosis

Variables	*n* = 63
Age (median, range)	66 (39–81)
BMI (median, range)	25 (16–36)
Gender	
Male	47 (65)
Female	16 (25)
Comorbidities	
Cardiac	16 (25)
Vascular	26 (41)
Diabetes mellitus	13 (21)
Pulmonary	11 (18)
ASA-score	
1	2 (3)
2	29 (46)
3	31 (49)
4	1 (2)
Neoadjuvant therapy	
Chemo(radio)therapy	57 (91)
None	6 (10)
Pathological T stage	
pT0	19 (30)
pT1	12 (19)
pT2	9 (14)
pT3	23 (37)

### Primary outcome

**Fig. 5:**
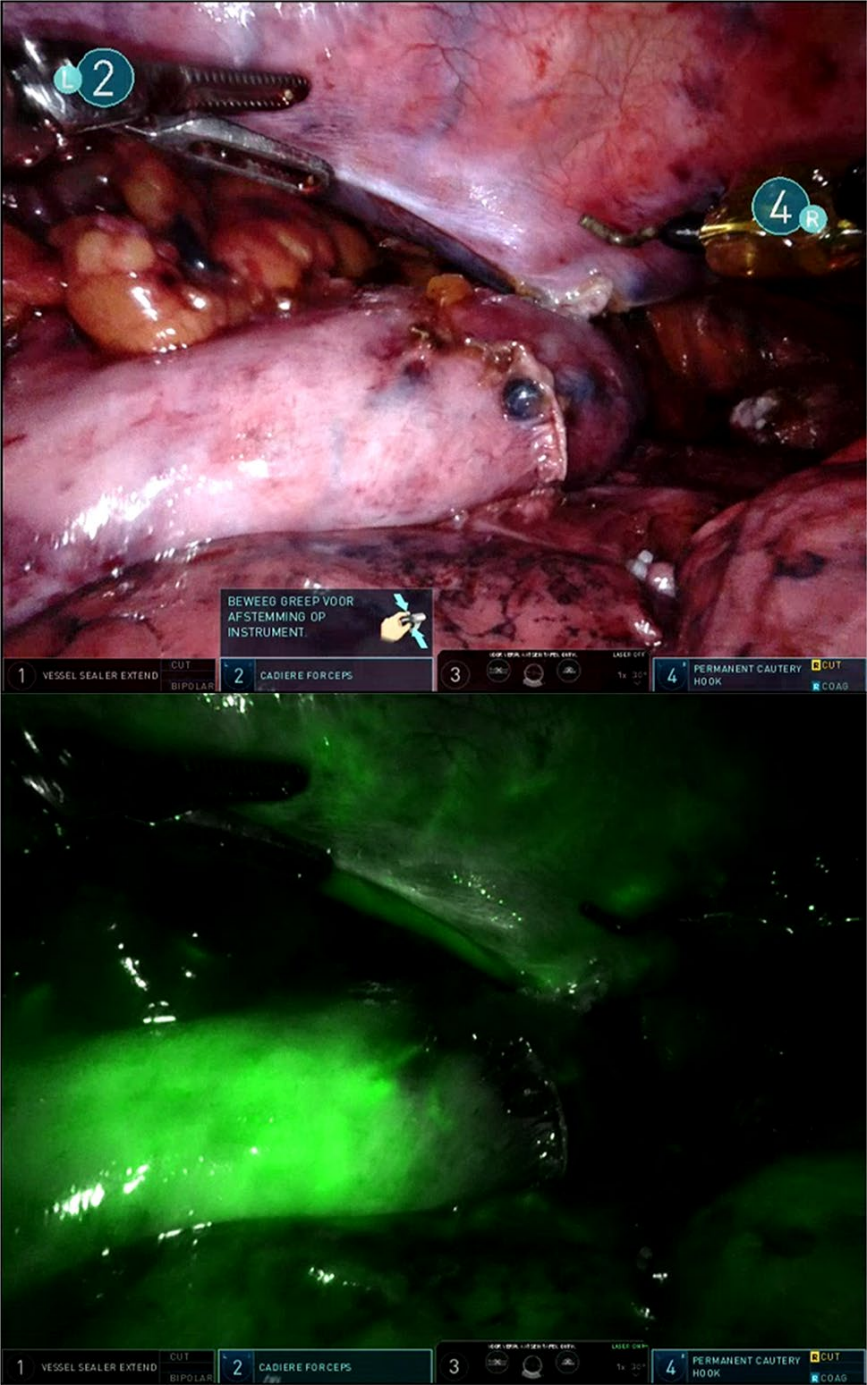
The tip of the gastric conduit did not enhance after administration of indocyanine green

Before ICG was administered, the planned site of the anastomosis was estimated at a median of 4 (range 1–9) cm measured from the tip of the gastric conduit (Table 2). This was done according to the subjective judgment of the surgeon, defining a well-perfused area without the use of ICG-FA. Based on subjective assessment of the ICG-FA signal, a change in the location of the planned anastomosis was deemed necessary in nine (14%) patients. An example is demonstrated in Fig. 5. This resulted in a shift in location of a median of 2 (range 0–3.5) extra cm towards the base. In these nine patients, the anastomosis was created at a median of 5 (range 2–9) cm from the tip of the gastric conduit. In one of the patients, a change of the anastomotic location was desired but was not possible because the gastric conduit did not have enough length. Out of the nine patients where ICG-FA resulted in a change of location of the anastomosis, three (33%) developed an anastomotic leakage. Out of the 54 patients in which the location of the anastomosis was not changed, 9 (20%) developed anastomotic leakage. There was no statistical difference in the development of anastomotic leakage after changing the location of the anastomosis according to ICG-FA (*P* = 0.403).

**Table 2:** Details on the anastomosis in 63 patients who underwent RAMIE with intrathoracic anastomosis

Variables	Total cohort, *n* = 63	Anastomotic leak, *n* = 14	No anastomotic leak, *n* = 49	*P* value
	Patients, *n* (%)			
Change in location of the anastomosis	9	3 (33)	6 (67)	0.403
No change in location of the anastomosis	54	11 (20)	43 (80)	
Distance between the tip of the gastric conduit and the anastomosis in cm, median (range)				
Before ICG was administered	4 (1–9)			
After ICG was administered	5 (2–9)

### Secondary outcome

The surgical video was not suitable for analysis in 35/63 patients. Reasons for exclusion were: movement of the robotic camera (*n* = 15), missing surgical video (*n* = 2), technical problem with the video server (*n* = 10), ROI not in 1 snapshot (*n* = 3). Finally, 28 RAMIE videos were suitable for ICG-FA quantification.

The lung was the first region of interest to reach its FI_max_, with a median *T*_max_ of 7 (range 3–36) seconds, followed by the thoracic wall (*T*_max_ 18 s, range 7–42). Subsequently, the gastric conduit reached maximum enhancement in a base-to-tip order, with inclining median Tmax the further the ROI was located away from the base (Table 3). Quantification of ICG was performed in 3 patients with anastomotic leakage and 25 patients without anastomotic leakage (Table 3). For patients with anastomotic leakage, the median time to reach FImax at the base was 26 s (range 24–40) compared to 23 s in patients without anastomotic leakage (*p* = 0.413). The median time to reach the FImax at the tip was 56 s (range 30–83) in patients with anastomotic leakage compared to 34 s (range 12–66) in patients without anastomotic leakage (*p* = 0.320).

**Table 3:** Quantification of ICG in 28 patients who underwent RAMIE with intrathoracic anastomosis

Region of interest (ROI)	Time to maximum intensity, s, median (IQR)			*P* value
	Total cohort, *n* = 28	Anastomotic leak, *n* = 3	No anastomotic leak, *n* = 25	
Gastric conduit				
Base	25 (13–49)	26 (24–40)	23 (13–49)	0.413
¼	33 (11–71)	38 (30–42)	24 (11–71)	0.297
½	35 (2–76)	35 (29–49)	28 (2–76)	0.480
¾	42 (14–76)	44 (22–72)	42 (14–76)	0.603
Tip	34 (12–83)	56 (30–83)	34 (12–66)	0.320
Lung	7 (3–36)	7 (5–21)	7 (3–36)	0.911
Thorax	18 (7–42)	15 (10–19)	19 (7–42)	0.296

## Discussion

This study prospectively investigated the effect of implementation of ICG-FA during RAMIE in 63 patients. Based on ICG-FA, the planned location of the anastomosis was changed in 9 (14%) patients. Overall, the time to maximum intensity of the base of the gastric conduit was shorter (median 25 s; range 13–49) compared to the tip of the gastric conduit (median 34 s; range 12–83). In patients with anastomotic leakage (*n* = 3), perfusion of the entire gastric conduit was worse compared to patients without anastomotic leakage (*n* = 25) as indicated by the time to maximum fluorescence intensity, although this was not statistically significant (*p* = 0.320). For patients with anastomotic leakage, the median time to reach FImax at the base was 26 s (range 24–40) and the tip 56 s (range 30–83). For patients without anastomotic leakage, this was median 23 s at the base and 34 s (range 12–66) at the tip.

Although the administration of ICG-FA before the creation of the anastomosis led to an adaptation of the anastomotic location in 9 (14%) patients, still 3 (33%) patients continued to developed anastomotic leakage. This is in line with the results of a recent study which reported a change in anastomotic location based on ICG-FA in 11 (13%) patients of which 5 (45%) developed anastomotic leakage [15]. Several factors may contribute to the persistent high incidence of anastomotic leakage despite changes in anastomotic location. First, this may represent a status of impaired perfusion of the entire gastric conduit leading to a high rate of anastomotic leakage regardless of the location of the anastomosis [16]. Second, a change in anastomotic site towards the base at the gastric conduit could have caused too much tension on the anastomosis and therewith hindering optimal healing of the anastomosis. On the other hand, the question whether changing the location of the anastomosis based on ICG-FA have actually resulted in the prevention of anastomotic leakage will remain unanswered as there was no formal control group in the current study design.

**Fig. 6:**
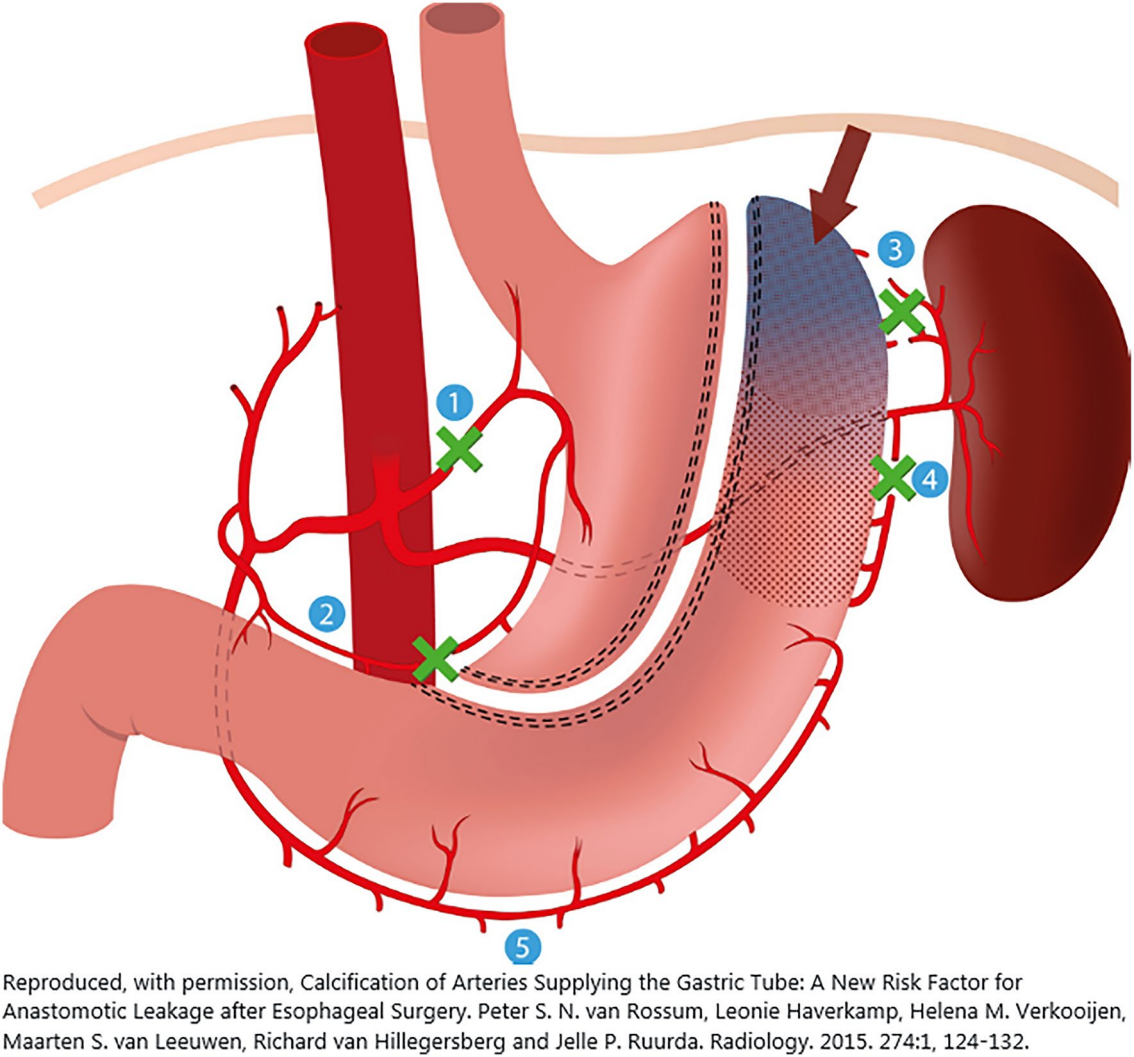
Arterial supply of the gastric conduit during esophageal surgery. The base of the gastric conduit is vascularized by branches of the right gastroepiploic artery, while the perfusion of the tip of the gastric conduit depends on intramural perfusion

A recent meta-analysis investigated if the use of ICG-FA resulted in a lower incidence of anastomotic leakage in patients who underwent minimally invasive Ivor-Lewis esophagectomy [17]. No difference in incidence of anastomotic leakage was observed. These results are conflicting with other studies and meta-analysis that did show a significant reduction in anastomotic leakage after esophagectomy with the use of ICG [18–20]. A possible explanation for these contradicting findings could be the difference between a cervical and intrathoracic anastomosis. Most studies included cervical anastomosis that are known to be made in a less perfused part of the conduit more often than intrathoracic anastomoses. This could be explained by the length and tension of the gastric conduit that is needed for a cervical anastomosis. This means that a longer part of the gastric conduit is dependent on intramural perfusion (Fig. 6) [21]. In conclusion, the added value of ICG-FA during esophagectomy with intrathoracic anastomosis might be inferior compared to cervical anastomosis.

In this study, surgical videos were reviewed to quantify ICG-FA and objectively asses the time to maximum fluorescence of the different ROI, which is considered the best quantification method [9]. The median time to maximum intensity was first reached by the lung and thoracic wall followed by the gastric conduit in base-to-tip direction. This could be explained by the anatomy of the human blood circulation and the direction of blood flow within the right gastroepiploic artery. Interestingly, in patients with anastomotic leakage it took longer for the ICG signal to reach its maximum intensity in the gastric conduit compared to patients without anastomotic leakage. Several other studies have measured the ICG signal at different ROI and compared the time to (maximum) intensity between patients with and without anastomotic leakage [15, 22]. Comparable to the current study, a longer time to (maximum) enhancement was observed for patients with anastomotic leakage. These findings support the hypothesis that patients with anastomotic leakage have an impaired perfusion of the entire gastric conduit.

Quantification of ICG-FA could be of added value in a standardized and reproducible research setting. However, the clinical relevance of quantifying ICG-FA is questionable. The base of the gastric conduit is likely to be better perfused than tip [8]. Hence, one could argue that the anastomosis should always be located as close to the base as possible, taking the right amount of tension into account. With this theory in mind, the added value of ICG-FA could be debated. If the anastomosis is located as close to the base as possible and ICG-FA still shows that the perfusion of the gastric conduit might not be sufficient, the options to prevent anastomotic leakage are very limited. Reported options are the pre-emptive placement of an endoscopic vacuum device or to create an esophagostomy and postpone continuity restoration [16]. These options are invasive and burdensome to patients and their effect ever so unpredictable. In addition, the assessment of ICG-FA should be substantially certain in predicting anastomotic leakage before considering any of these options. To date, no validated cut-off values with such a high predictive value exist. Besides preventing anastomotic leakage, ICG might help to identify patients at risk for anastomotic leakage which could create extra awareness during the postoperative course. These patients could be subjected to an intensified postoperative monitoring with a low threshold for performing diagnostic imaging or an endoscopy. In addition, extended nil per mouth policy or prolonged observation in hospital could be considered. However, this will not prevent the anastomosis from leaking.

Instead of diagnosing a perfusion problem intraoperatively during esophagectomy, identifying patients at risk prior esophagectomy would be of greater value. This includes a more detailed assessment of the vascular status of patients followed by implementation of preventative measures. Currently, the ISCON trial is an example of such an attempt to investigate this option [23]. In that study, selected patients undergo laparoscopic ischemic precondition 12–18 days prior esophagectomy. Patients are selected based on calcifications in the thoracic aorta according to the Uniform Calcification Score or the celiac trunk according to the NASCET score [24, 25]. Laparoscopic ischemic preconditioning consists of the transection of the left gastric artery and the short gastric arteries prior esophagectomy in order to stimulate a redistribution of the blood flow for the future gastric conduit. Results of ISCON are promising, but more studies are needed to demonstrate the efficacy [26].

Strength of the current study is the prospective design with a predefined standardized procedure for ICG-FA. In addition, a truly objective and an easy reproducible method to quantify ICG-FA was used and subjective factors were excluded. However, several limitations apply to this study as well. First, quantification could only be performed in 28 of the 63 patients. A major reason for exclusion was movement of the robotic camera during the ICG assessment which is likely caused by a learning curve effect as the importance of a fixed snapshot was appreciated later during the study. This is supported by the fact that almost all videos with movement were from patients who underwent RAMIE within the first of this study. Another reason for the missing videos where the technical problems with the storage of the surgical videos prohibiting it from analyses. These issues lead to the fact that we only had three patients with anastomotic leakage that could be evaluated by quantified ICG-FA. An appropriate correlation between quantified ICG-FA and anastomotic leakage is, therefore, not possible.

The incidence of anastomotic leakage was relatively high in this cohort (22%). This is most likely caused by a learning curve effect for the hand-sewn robot-assisted intrathoracic anastomosis, that has been published previously [27]. In the most recent 1.5 years, our leak rate has decreased to 6%, indicating that we have reached proficiency in the past 2 years following this cohort.

In this prospective study, the use of ICG-FA resulted in an adaptation of the anastomotic site in nine (14%) patients during RAMIE with intrathoracic anastomosis. In addition, the quantification of ICG-FA showed that the gastric conduit reached its maximum intensity in a base-to-tip direction. Perfusion of the entire gastric conduit was worse for patients with anastomotic leakage.

## References

[1] Shapiro J, van Lanschot JJB, Hulshof MCCM (2015). Neoadjuvant chemoradiotherapy plus surgery versus surgery alone for oesophageal or junctional cancer (CROSS): long-term results of a randomised controlled trial. Lancet Oncol.

[2] Low DE, Kuppusamy MK, Alderson D (2019). Benchmarking Complications Associated with Esophagectomy. Ann Surg.

[3] Griffiths EA (2021). Rates of Anastomotic Complications and their Management following Esophagectomy. Ann Surg.

[4] Goense L, van Dijk WA, Govaert JA (2017). Hospital costs of complications after esophagectomy for cancer. Eur J Surg Oncol.

[5] Goense L, van Rossum PSN, Tromp M (2017). Intraoperative and postoperative risk factors for anastomotic leakage and pneumonia after esophagectomy for cancer. Dis Esophagus.

[6] Borggreve AS, Goense L, van Rossum PSN (2018). Generalized cardiovascular disease on a preoperative CT scan is predictive for anastomotic leakage after esophagectomy. Eur J Surg Oncol.

[7] Boyle NH, Pearce A, Hunter D (1998). Scanning laser Doppler flowmetry and intraluminal recirculating gas tonometry in the assessment of gastric and jejunal perfusion during oesophageal resection. Br J Surg.

[8] Schilling MK, Redaelli C, Maurer C (1996). Gastric microcirculatory changes during gastric tube formation: assessment with laser Doppler flowmetry. J Surg Res.

[9] Slooter MD, Mansvelders MSE, Bloemen PR (2021). Defining indocyanine green fluorescence to assess anastomotic perfusion during gastrointestinal surgery: systematic review. BJS open.

[10] de Groot EM, Kingma FB, Goense L (2021). Robot-assisted hand-sewn intrathoracic anastomosis after esophagectomy. Ann Esophagus.

[11] van der Sluis PC, Ruurda JP, van der Horst S (2012). Robot-assisted minimally invasive thoraco-laparoscopic esophagectomy versus open transthoracic esophagectomy for resectable esophageal cancer, a randomized controlled trial (ROBOT trial). Trials.

[12] van der Sluis PC, Ruurda JP, van der Horst S (2018). Learning curve for robot-assisted minimally invasive thoracoscopic esophagectomy: results from 312 cases. Ann Thorac Surg.

[13] Koyanagi K, Ozawa S, Oguma J (2016). Blood flow speed of the gastric conduit assessed by indocyanine green fluorescence: new predictive evaluation of anastomotic leakage after esophagectomy. Med (United States).

[14] Low DE, Alderson D, Cecconello I (2015). International consensus on standardization of data collection for complications associated with esophagectomy: esophagectomy complications consensus group (ECCG). Ann Surg.

[15] Slooter MD, de Bruin DM, Eshuis WJ (2021). Quantitative fluorescence-guided perfusion assessment of the gastric conduit to predict anastomotic complications after esophagectomy. Dis Esophagus.

[16] Slooter MD, Eshuis WJ, Cuesta MA (2019). Fluorescent imaging using indocyanine green during esophagectomy to prevent surgical morbidity: a systematic review and meta-analysis. J Thorac Dis.

[17] Casas MA, Angeramo CA, Bras Harriott C (2021). Indocyanine green (ICG) fluorescence imaging for prevention of anastomotic leak in totally minimally invasive Ivor Lewis esophagectomy: a systematic review and meta-analysis. Dis Esophagus.

[18] Ladak F, Dang JT, Switzer N (2019). Indocyanine green for the prevention of anastomotic leaks following esophagectomy: a meta-analysis. Surg Endosc.

[19] Zehetner J, DeMeester SR, Alicuben ET (2015). Intraoperative assessment of perfusion of the gastric graft and correlation with anastomotic leaks after esophagectomy. Ann Surg.

[20] Koyanagi K, Ozawa S, Ninomiya Y (2021). Indocyanine green fluorescence imaging for evaluating blood flow in the reconstructed conduit after esophageal cancer surgery. Surg Today.

[21] Van Workum F, Verstegen MHP, Klarenbeek BR (2021). Intrathoracic vs cervical anastomosis after totally or hybrid minimally invasive esophagectomy for esophageal cancer: a randomized clinical trial. JAMA Surg.

[22] Talavera-Urquijo E, Parise P, Palucci M (2020). Perfusion speed of indocyanine green in the stomach before tubulization is an objective and useful parameter to evaluate gastric microcirculation during Ivor-Lewis esophagectomy. Surg Endosc.

[23] Veen A van der, Schiffmann LM, de Groot EM, et al (2022) The ISCON-trial protocol: laparoscopic ischemic conditioning prior to esophagectomy in patients with esophageal cancer and arterial calcifications. BMC Cancer. 10.1186/s12885-022-09231-x 10.1186/s12885-022-09231-x PMC8817569 35123419

[24] Van Rossum PSN, Haverkamp L, Verkooijen HM (2015). Calcification of arteries supplying the gastric tube: a new risk factor for anastomotic leakage after esophageal surgery. Radiology.

[25] Chang DH, Brinkmann S, Smith L (2018). Calcification score versus arterial stenosis grading: comparison of two CT-based methods for risk assessment of anastomotic leakage after esophagectomy and gastric pull-up. Ther Clin Risk Manag.

[26] Michalinos A, Antoniou SA, Ntourakis D (2020). Gastric ischemic preconditioning may reduce the incidence and severity of anastomotic leakage after οesophagectomy: a systematic review and meta-analysis. Dis Esophagus.

[27] de Groot EM, Möller T, Kingma FB, Grimminger PPTB, van Hillegersberg R, Jan-HendrikEgberts JPR (2020). Technical details of the hand-sewn and circular-stapled anastomosis in robot-assisted minimally invasive esophagectomy. Dis Esophagus.

